# A true case story

**DOI:** 10.3402/mehd.v23i0.18985

**Published:** 2012-08-24

**Authors:** Hanne Bjørg Walker

**Affiliations:** BioMed Clinic, Oslo, Norway

**Keywords:** Autism, child, vaccination, muscle tone, dietary intervention, gluten free diet, sorghum

## Abstract

Autism is not generally recognized as a condition which can be bio-medically influenced. As of today, there are no biomarkers for autism that are recognized by traditional medicine. Treating autism medically is a difficult and hopeless task according to official guidelines (even though it is seldom written in official documents). Parents of many children who have or had an ASD diagnosis have witnessed significant improvements in their children after dietary interventions as well as after interventions with vitamins, minerals and biogene substances which can be bought over the counter. The parents of individual children are their best observers. With a high degree of certainty, they are able to see which substances improve or weaken their children. Their observations are usually accurate, but their rationale for why is often wrong. Observations from parents can often be of greater importance for the child than advice from so called experts.

This is a true story about a girl whose parents lost contact with her when she was only 6 months old. In her first 14 days she lost her ability to roll over, to babble and make sounds. She did not look at her parents any more – just stared up at the roof. At 9 months she did not respond to words such as, ‘look at mommy’. Through the parents own experiences with her older siblings and 4 months of frantic searching for a diet that would agree with the child, she made a remarkable journey from 10 months of age to 18 months. There is one thing worth mentioning – she refused to eat solid food throughout this time.

The story does not end there. Today she is 12 years old and has had to be regulated with diet and biogene substances every day since she was 4 years old. During the last 5 months she has shown more stability and can even go a day or two without biogene substances as long as she keeps to her dietary plan. If you had just met her and spent a day with her, you would never know.

The case presented is as follows: Mother Norwegian, father US citizen. Pregnancy and delivery normal. Developed normally up to 5½ months of age. Could roll over, make sounds, cooing and gurgling, loving eye contact. First vaccination (DTP, polio, Hib) at 3 months of age, no reaction except oral thrush. Same vaccines at 5½ months. Within 14 days she lost the ability to roll over, she did not coo any more and had a blank stare.

Experience of two siblings who also have some ADS symptoms made the girl's parents switch to a diet based on rice and water. Within a week she regained eye contact but she did not regain her ability to roll over and make cooing or gurgling sounds.

Her muscle tone was very weak for the next 4 months. She could not sit by herself unsupported. She did not respond to sounds and she did not respond when sitting on her mother's lap to doctors asking her to look at mommy, where is mommy, etc.

Reports from professionals stated that at 9 months of age she was withdrawn, uninterested in other children, had very weak muscle tone, uninterested in food and liked best to sit in a reclining chair with a blank stare. She made very little noise but cried approximately 10 to 20 min after eating. She was also totally uninterested in toys. Her comfort during these 4 months was to be carried and held closely – which she was – almost 24 hours a day.

During the period from 6 to 10 months the parents did a frantic search for a diet that was compatible with her. They did a search for a nutritional formula in Europe and the USA, trying every product available. She did not tolerate any of them. She reacted most negatively to a milk-based supplement and even more to Peptide 0–2 from SHS International Ltd., Liverpool, UK – a complete nutritional support for children with impairment of the gastrointestinal tract. This formula contained hydrolyzed protein from meat and soya, refined animal fat from pork, and peanut oil among other ingredients.

The last supplement she tried at 10 months old was the complete hypoallergenic powdered infant feed consisting of essential and non-essential amino acids, carbohydrate, fat, minerals, trace elements and vitamins – Neocate 0–12 months from SHS International (Liverpool, UK) (Table 1). Within 2 days she was different. Over the next 8 months (from 10–18 months of age) she had virtually no other food. She recovered totally.

Her recovery at age 18 months is fully documented in papers from pediatricians. She did not have any examination done of her intestine at that time. At 2 years of age her cognitive functions were excellent and her motor skills impressive.

At age two – still refusing to eat solid food – her parents switched her supplementary infant feed to Neocate Advance, unflavored. She was given extra Cod Liver Oil from age 1 and calcium from age two. She remained on Neocate Advance from 2 to 4 years old. In this period she had a very slow regression. Her regression was so slow that her parents did not notice it at first.

At age three she started pressing her eyes with her fingers. She could also explain that her eyes hurt. Thorough investigation of her eyes did not reveal any problems. Due to earlier reports her parents doubled the amount of her calcium supplement. Within 2 weeks her eye problems disappeared.

During this period of slow regression she started urinating frequently during the day. Bacteriological examinations revealed sterile urine. She needed to go to the toilet every time she started a meal. Even sitting down at the dinner table with her siblings triggered this.

At age four an endoscopy was performed in New York, demonstrating a mild terminal ileitis and right-sided colitis. Long-term therapy with balsalazide initiated a slow improvement in behavior and a similar improvement in eating habits. Stopped after 6 months because of experience with siblings on same drug and possible side effects on mineralization of bone.

**Table 1:** Ingredients in dietary supplements

Compound	Neocate 0–12 months per 100 g	Neocate Advanced (unflavoured) per 100 g
**Amino acid profile g**		
L-Alanine	0.61	0.39
L-Arginine	1.08	1.76
L-Aspartic acid	1.01	0.71
L-Cystine	0.4	0.04
L-Glutamic A	nil added	nil added
Glycine	0.95	0.63
L-Histidine	0.62	0.46
L-Isoleucine	0.95	0.63
L-Leucine	1.63	1.06
L-Lysine	1.11	0.78
L-Methionine	0.26	0.5
L-Phenylalanine	0.73	0.88
L-Prolin	1.16	0.74
L-Serine	0.71	0.46
L-Threonin	0.8	0.53
L-Tryptophan	0.32	0.21
L-Tyrosine	0.73	0.18
L-Valine	1.04	0.67
L-Carnitine	0.01	11.17
Taurin	0.02	23.33
L-Glutamine	1.34	1.38
**Vitamins**	per 100 g	
Vitamin A mcg	528	148
Vitamin D mcg	8.5	3.24
Vitamin E mg	4.5	2.3
Vitamin C mg	40	13.2
Vitamin K mcg	21	6.3
Thiamin mg	0.39	0.22
Riboflavin mg	0.6	0.26
Niacin mg	4.5	3.8
Vitamin B6 mg	0.52	0.32
Folacin mcg	38	28
Vitamin B12 mcg	1.25	0.28
Biotin mcg	26	8
Pantothenic Acid mg	2.65	1
Choline mg	50	76.8
Inositol mg	100	7.56
**Minerals**	per 100 g	
Sodium mg	120	240
Potassium mg	420	468
Chloride mg	290	368
Calcium mg	325	180
Phosphorus mg	230	140
Magnesium mg	34	50
**Trace elements**	per 100 g	
Iron mg	7	2.48
Copper mg	0.38	0.18
Zinc mg	5	1.76
Manganese mg	0.38	0.14
Iodine mcg	47	28
Molybdenum mcg	14.25	7.4
Selenium mcg	11	9.2
Chromium mcg	10	4.92
**Typical fatty acid profile**	g per 100 g fatty acid	
C6	0.15	1
C8	2.1	21
C10	2.4.	15
C12	14.4	1
C14	4.8	trace
C16	7.33	3
C16:1	1.1	nill
C18	0.83	2
C18:1	47.37	42
C18:2	17.53	11
C18:3	1.73	3
C20		trace
C20:1		trace
C22:1		<1

Amounts as labeled on boxes, produced 2001–2004 and in brochure from SHS, same period.

Fava is a species of family Fabaceae, subfamily Faboideae, tribe Vicieae, genus Vicia.

Teff is a species of family Poaceae, subfamily Chloridoideae, genus Eragrostis (lovegrass).

Quinoa is a species of family Amaranthaceae, subfamily Chenopodioidea, genus Chenopodium (goosefoot).

Buckwheat is a variety of plants in the dicot family Polygonaceae, genus Fagopyrum.

Amaranth is a species of family Amaranthaceae, subfamily Amaranthoideae, genus Amaranthus.

Sorghum is a species of family Poaceae, subfamily Panicoideae, tribe Andropogoneae, genus Sorghum.

Garbanzo or chickpea is a species of family Fabaceae, genus Cicer.

As stated above, eating solid food was not easy for her. Due to her sibling's history and experience she was offered for the first time at age four a gluten free bread made from fava flour which naturally contained L-Dopa, together with rice flour and garbanzo flour.

She accepted this bread and had no adverse reaction to it. She has been eating the same bread for 8 years. She has tried other flour during this period, none of which are compatible with her for more than a day or two. She regresses slowly on small amounts of sorghum. She reacts immediately within hours or days to bread containing small amounts of the following flours: teff, quinoa, amaranth, buckwheat, soya, corn and wheat starch. All of them trigger or have triggered in the past years a shift in her daily appearance such as the size of her pupils, watery eyes, unnatural posturing, etc.

Neurofeedback twice weekly for 1½ years and Tomatis for 60 sessions was an important intervention for her – starting a year after her second regression together with her dietary changes. These sessions always made her hungry.

From age 4 and up to last year she refused to eat chicken or pork. Besides gluten free bread, she eats moose/elk, reindeer, grass fed beef, vegetables low in oxalate, fruits and berries. No fish.

Other than her diet, daily observation and daily regulation of her with supplements have been the most important tools.

In addition to the immediate reactions from food, her parents have also seen changes in the protrusion of her ears, and heart palpitation when lying down. Her ability to tell how she feels has been of great help to her parents in modifying her diet and supplements – as have their experiences with her older siblings. Today she is a well-functioning young lady.

Over the years I have had the privilege of meeting a number of couples with an autistic child. Based upon their information and the present case report I am prepared to make the following statement:

## Main message to parents

Treatment of an autistic child is a year-long experience in trials, errors and hopefully, success. It is best to remember the following:

1. You are your child's best observers. Trust your own observations.
2. Each child has to be treated on an individual basis.
3. In addition to love, dietary interventions are by far the most important factors for success.
4. Autism is treatable.

